# Building Bridges between Theory and Practice: How Citizen Science Can Bring Equine Researchers and Practitioners Together

**DOI:** 10.3390/ani10091644

**Published:** 2020-09-13

**Authors:** Kate Fenner, Katherine Dashper, Cristina Wilkins, James Serpell, Andrew McLean, Bethany Wilson, Paul McGreevy

**Affiliations:** 1 Sydney School of Veterinary Science, University of Sydney, Camperdown, NSW 2006, Australia; bethany.wilson@sydney.edu.au (B.W.); paul.mcgreevy@sydney.edu.au (P.M.); 2 School of Events, Tourism and Hospitality Management, Leeds Beckett University, Leeds LS1 3HE, UK; K.Dashper@leedsbeckett.ac.uk; 3 Saddletops Pty Ltd., Gatton, QLD 4343, Australia; cristinaluz@horsesandpeople.com.au; 4 School of Veterinary Medicine, University of Pennsylvania, Philadelphia, PA 19104, USA; serpell@vet.upenn.edu; 5 Equitation Science International, 3 Wonderland Ave, Tuerong, VIC 3915, Australia; andrewmclean@esi-education.com

**Keywords:** online survey, equine behaviour monitoring, welfare, rider safety, motivating stakeholders, domestic equine triad

## Abstract

**Simple Summary:**

Horse owners and caregivers are ideally placed to inform equine researchers about the training, management and behaviour of their horses, and online surveys are a simple and easy way to collect this information. However, as potential survey respondents may need some incentive to engage in detailed surveys, we investigated the popularity of four potential no-cost tools to compensate survey participants for their time. While our inquiry found at least one of these tools would be of benefit to most participants, it also revealed the need to improve horse riders’ and handlers’ access to scientific findings.

**Abstract:**

Over the last decade, equitation scientists have increasingly relied on online survey tools to gather information on horse training, management, behaviour and other equine-related subjects. With a detailed knowledge of their animals, horse owners and riders are ideally placed to contribute to research but are sometimes reluctant to engage with and devote time to surveys. The current article reveals, through consultation with stakeholder groups, the potential of a range of motivational items to boost horse-owner participation. A short, three-question inquiry was developed to rank respondents’ (*n* = 747) preferred survey tools and other items designed to engage the equestrian community with the donation of data. Respondents were asked to assign themselves to one of four categories: academics/researchers, professionals, practitioners and enthusiasts. The inquiry offered respondents the choice of three hypothetical tools: a standardised tool to measure behaviour over time; a logbook tool to record training and behaviour on a regular basis; and a chart to compare an individual horse’s behaviour with that of the general horse population. While analysis revealed that stakeholders considered at least one of the tools to be useful, it also exposed significant differences among the perceived usefulness of the various tools themselves. Using free-text responses, participants described the challenges faced when gathering information on horse training, management and behaviour. Qualitative analysis of these data revealed the need to improve the current dissemination of scientific findings to bridge various knowledge gaps. The Equine Behavior Assessment and Research Questionnaire (E-BARQ) is a longitudinal instrument that investigates horse training and management practices and permits an analysis of their relationship with behaviour. The current stakeholder consultation contributed to the final version of the E-BARQ questionnaire, identified incentivising items that can be offered to putative E-BARQ respondents, guided the eventual selection of a *Share-&-Compare* feedback chart, and reinforced the need for open-access dissemination of findings.

## 1. Introduction

Horse behaviour and the bond formed between horses and humans have been the subjects of fascination since ancient times. Modern equitation and husbandry approaches are a mix of myth and useful practice techniques that have developed over centuries. The ways we train and manage horses are reflected in and reflective of their behaviour [1], which in turn, affects horse welfare and human safety. For example, behavioural issues are considered the main reason for wastage among riding horses, where horses are sold-on or euthanised because they become difficult to handle or ride, thereby jeopardising handler and rider safety [2]. In contrast, improved horse management and training practices increase the prevalence of desirable behaviour and positive welfare outcomes [3,4]. However, to make meaningful and enduring inroads into improving rider safety and horse welfare, we first need to understand how horses’ current and historic training and management practices influence behavioural responses.

Equitation science is working to identify training and management practices that optimise welfare and performance in ridden horses. However, research into horse training, management and behaviour can be prohibitively expensive. Small-scale studies, comparing one training or management method with another [5] are useful, but often have a narrow focus [6]. These factors of scale and focus combine to limit the generation of helpful, evidence-based, practical applications for horse riders and handlers. In contrast, large-scale data, of over 2000 responses, on current behaviour, training and management practices across various equestrian disciplines, horse breeds and countries can reveal how to improve both human safety and horse welfare. Collecting such data is challenging, since many horse owners operate in isolation from one another and may have had minimal experience in horse-keeping.

Considerable research has focused on professional and elite-level riders and horses [7], yet most domesticated horses are under the care of the recreational rider/owner group and few studies have established the needs of this important demographic [8]. Targeting this group of horse owners and riders through large-scale research projects can provide valuable insights and, in addition, could engage this grass-roots demographic with evidence-based horse training and management, leading to improved practice.

An increasingly popular way to collect large-scale data from a diverse demographic is through citizen science projects of the type known as contributory projects, that is, those based on Hecht and Rice’s [9] Model 2, where citizens provide unanalysed raw data. This model has the benefit of giving researchers direct access to participant-acquired, easily accessible data gathered from a questionnaire suitable for mobile devices or desktop computers. The Equine Behavior Assessment and Research Questionnaire (E-BARQ), a global horse training, management and behaviour database, is an example of one such project. E-BARQ is designed to measure elements of the domestic equine triad of training, management and behavioural parameters over time. As such, E-BARQ serves as a first step toward defining and incorporating good equine training, mental state, housing and behaviour.

The questionnaire, which is accessible to all horse owners or caregivers, builds on the Canine Behavior Assessment Research Questionnaire (C-BARQ), the canine version of a similar project, that was launched in 2005, and has since collected data on over 85,000 dogs and been used in more than 100 published research studies. When compared to their canine equivalents [10], standards of horse welfare remain discouragingly low. However, both species can suffer from owners’ lack of knowledge on the importance of evidence-based practice [11,12].

Participation in large-scale data collection projects and citizen science can broaden the public’s understanding of the scientific process and facilitate the dissemination of research findings [9]. As the E-BARQ survey is a longitudinal study, respondents can begin to appreciate how changes in their training and management, in combination, influence their horse’s behaviour and performance over time. Meanwhile, with large numbers of horses being trained and managed in various ways, it is anticipated that interrogation of the E-BARQ dataset will reveal the role of various common training and management interventions as risk factors for undesirable behaviour. It will also reveal early behavioural predictors of undesirable behaviours, thus improving rider safety by permitting remediation, where appropriate.

One of the challenges of this type of data collection is participant completion rates, particularly when lengthy questionnaires are involved. This obstacle may be overcome by offering users an incentive in the form of feedback on their contribution [9]. When collecting data on equine and equitation science, an incentive item could take the form of feedback on the owner’s horse compared to the population, such as that offered by the E-BARQ *Share-&-Compare* graph. Additionally, such feedback can encourage users to repeat the questionnaire at regular intervals to monitor improvement upon their previous results.

Despite its advantages over short surveys, the E-BARQ questionnaire, with 97 questions, is a relatively lengthy instrument, taking 20 to 30 min to complete. Data collection via online surveys has become increasingly common over the last two decades [13], with the development of numerous survey platforms (such as Survey Monkey, Google Forms, Zoho Survey, Survey Gizmo and Survey Planet), leaving some potential respondents disinclined to engage with lengthy surveys [14], let alone complete them [15]. In this context, the success of contributory projects, such as the E-BARQ platform, may be boosted if they offer potential respondents some form of reward or incentive for engaging with the survey [16]. The C-BARQ survey (www.cbarq.org), for instance, provides each dog owner with a color-coded chart that plots their dog’s behavioural scores against the average scores for the dog population as a whole. Similarly, the E-BARQ is designed to incorporate these kinds of non-financial inducements to encourage participation from stakeholders.

This article explores, through consultation with stakeholder groups, strategies to motivate horse-owning respondents to donate their data. The primary goal of the current study was to gauge how likely stakeholders from various groups would be to engage with E-BARQ when one or more motivational items were included to entice respondents to contribute their data.

## 2. Materials and Methods

A short, three-question, online inquiry was designed using Bucket.io (Bucket.io, Georgetown, TX, USA) to gather information from equine stakeholders. The inquiry link was distributed primarily on *Facebook*, but also shared on Instagram, Twitter and LinkedIn; targeting general horse groups in addition to equine and equitation science groups. Participants were encouraged to share the inquiry link with their horse-riding associates. The link was also distributed to the Kandoo Equine and Horses & People Magazine electronic newsletter subscribers. The inquiry was completed anonymously, and no demographic data was collected. Distribution was expected to attract English language respondents, but was not confined to a specific demographic.

Four equine stakeholder groups were targeted: (a) academics/researchers, (b) professionals (veterinarians and behaviourists), (c) practitioners (instructors and coaches) and (d) enthusiasts (owners, breeders and riders). As many equestrians fit into more than one of these groups, respondents were asked to nominate the stakeholder group that best described them.

### 2.1. Closed-Ended Responses

Stakeholder groups were offered only those incentive items that were appropriate and relevant for their chosen group. For example, the E-BARQ database, a repository that academics might wish to contribute to and use in their research, was not offered to practitioners or enthusiasts, as it would not be made available for such a purpose. In contrast, the *Share-&-Compare* graph was considered a potentially useful item for the professional, practitioner and enthusiast stakeholder groups. The study directed self-selected stakeholder respondents to the following question-and-answer choices:

(a) Academics were asked: when it comes to gathering or accessing large scale data on horse behaviour and training, which one of the following would be most useful to you?

Response options were: (1) a standardised, wide-ranging behavioural assessment tool (E-BARQ questionnaire alone); (2) an accessible data repository that I could both contribute to and use (E-BARQ database); and (3) honestly, neither of the above.

(b) Professionals were asked: when it comes to investigating or accessing large scale data on horse behaviour and training, which one of the following would be most useful to you?

Response options were: (1) a data repository that I could access to improve the service I provide to my clients (E-BARQ database); (2) somewhere to compare a particular client’s horse’s behaviour with other horses (*Share-&-Compare* graph); (3) somewhere to find information about behavioural and temperament traits (peer-reviewed reference library); and (4) honestly, none of the above.

(c) Practitioners were asked: when it comes to investigating scientific findings on horse behaviour and training, which one of the following would be most useful to you?

Response options were: (1) somewhere I/my clients could record a horse’s behaviour over time (every 6-months) (E-BARQ questionnaire); (2) somewhere I/my clients could keep regular (daily or weekly) records of my horses’ training and behaviour (*horselogbook*); (3) somewhere for my clients (and me) to compare a particular horse’s behaviour with others (*Share-&-Compare* graph); and (4) honestly, none of the above.

(d) Enthusiasts were asked: when it comes to learning about horse behaviour and training, which one of the following would be most useful to you?

Response options were: (1) somewhere I could record a horse’s behaviour over time (every 6-months) (E-BARQ questionnaire alone); (2) somewhere I could keep regular (daily or weekly) records of my horses’ training and behaviour (*horselogbook*); (3) somewhere to compare my horse’s behaviour with others (*Share-&-Compare* graph); and (4) honestly, none of the above.

#### Statistical Analysis

Pearson’s chi-square tests were performed to determine statistical significance between stakeholder choices of motivational items. Further post-hoc tests for pairwise independents were performed using the Holm method to correct the family-wise error rate. 

### 2.2. Open-Ended Responses

The survey provided all participants with the option to leave an open-ended free-text response describing their single biggest challenge or frustration when attempting to gather or access data and learn about horse behaviour and training.

The inquiry was available online from May to August 2018. Data were exported to Microsoft Excel 2016 (Redmond, WA, USA) and descriptive statistics reported at the International Society for Equitation Science conference, Rome 2018. The study was conducted under the approval of the University of Sydney Human Research Ethics Committee (approval number: 2012/656).

#### Qualitative Analysis

The qualitative responses were grouped by stakeholder group and then analysed thematically. To reduce subjectivity and the possibility of bias, responses were originally coded into themes by one researcher (KF) and analysed by a second research group member (KD). Open coding was performed initially within each stakeholder group to identify a broad range of themes, which were subsequently grouped to form overarching themes for each group. Although there were some similarities between different stakeholder groups, the ways in which respondents expressed their answers and the language used differed, as discussed further below. As a result, separate themes for each stakeholder group were maintained to reflect pivotal differences in emphasis and expression.

## 3. Results

The inquiry drew responses from 747 participants. Of these respondents, 11% (*n* = 83) identified primarily as academics/researchers, 10.5% (*n* = 78) as professionals, 18.3% (*n* = 137) as practitioners and 60.2% (*n* = 449) as enthusiasts. 

When asked about the perceived usefulness of the offered tools, stakeholder groups varied in their choices (see Table 1). Respondents were able to select ‘none of the above’ and 13.4% of academics indicated that none of the tools would be useful, 6.3% of professionals, 19.3% of practitioners and 31.4% of enthusiasts also indicated that none of the tools would be useful (see Table 1).

### 3.1. The Tools Offered

#### 3.1.1. The E-BARQ Database

The E-BARQ database was the most popular selection for both the academic and professional groups, although not significantly more popular than the next most popular section for either academics (proportion z test χ^2^ = 0.000, *p* > 0.999) or Professionals (χ^2^ = 3.227, *p* = 0.072).

#### 3.1.2. The E-BARQ Questionnaire

The E-BARQ questionnaire was offered as a behavioural evaluation tool to academics (perceived as most useful by 35 of 82) and as a means of tracking an individual horse’s behaviour over time to practitioners (perceived as most useful by 8 of 145) and enthusiasts (perceived as most useful by 54 of 544). Thus, academics rated the standardised behavioural assessment tool as the most useful significantly more often than practitioners (proportion z test χ^2^ = 44.731, *p* < 0.001) or enthusiasts (χ^2^ = 60.036, *p* < 0.001), to whom the tool was presented as a means of monitoring behaviour of a single horse longitudinally, at intervals of 6 months.

#### 3.1.3. The *Share-&-Compare* Graph

The *Share-&-Compare* graph was perceived as most useful by 133 of 544 (24.4%) enthusiasts, 35 of 145 (24.1%) practitioners, and 15 of 80 (18.8%) professionals. While professionals were less likely to find this tool most useful compared with access to the E-BARQ database (proportion z test χ^2^ = 11.513, *p* < 0.001), practitioners (test χ^2^ = 18.458, *p* < 0.001) and enthusiasts (χ^2^ = 39.287, *p* < 0.001) expected this prospect more appealing than the 6 monthly longitudinal monitoring of a horse’s behaviour over time using the E-BARQ tool, suggesting this group anticipate more benefits from comparing a horse to its peers than to its past self.

#### 3.1.4. The *Horselogbook*

The *horselogbook* was the tool that both groups perceived as most useful; 186 of 544 enthusiasts (34.2%) and 74 of 145 Practitioners (51.0%) respectively. For enthusiasts, the *horselogbook* was the only tool perceived as most useful more often than any of the other options (*Share-&-Compare* 24.7%, E-BARQ questionnaire alone 9.5% and none of the above 31.6%), although not significantly so (proportion z test χ^2^ = 0.817, *p* = 0.366). However, for practitioners, this opportunity for short-term longitudinal monitoring was perceived as significantly more useful than comparisons of a horse to its peers by *Share-&-Compare* (proportion z test χ^2^ = 21.226, *p* < 0.001).

### 3.2. Open-Ended Responses

The free-text box resulted in numerous responses that reflect the different levels of knowledge and varying interests and motivations of the different stakeholder groups. Within the academic group, 64.5% (*n* = 49) of respondents left a text response. The most prevalent themes for the academics were accessibility-of-research/findings (27.5%); credibility-of-findings (27.5%); and concerns-with-study-design/quality (22.5%). The academics were concerned by factors relating to study design and quality, notably small sample sizes in many published articles and inconsistency in methods and terminology used which limits potential for comparisons among studies, as well as the (scientific) reputations of some authors within the field. Within the professional group, 58.8% (*n* = 68) of respondents left a text response. The dominant themes were accessibility-of-research/findings (27.5%); credibility-of-findings (27.5%); and concerns-with-study-design/quality (22.5%). The professional group lamented the lack of open access to research reports and the dominant focus on English language-regions. Concern was expressed in 37.5% (*n* = 15) of text responses from professionals about the prevalence of anecdotal ‘evidence’ that lacks credibility and scientific underpinning. 

Within the practitioner group, 58.2% (*n* = 71) made free-text responses. The dominant themes were accessibility-of-research/findings (33.8%); concerns-with-study-design/quality (15.5%); and credibility-of-findings (15.5%). This group often reported that the language of academic articles was inaccessible to them and struggled to differentiate between scientifically informed, credible evidence and more anecdotal insights. They expressed concern that much horse-related research is ‘biased’ due to commercial funding and questioned the limitations of scientific evidence to really ‘know’ what a horse may think or feel, stressing the importance of acknowledging the individuality of horses in addition to shared characteristics and experiences. 

Finally, within the enthusiast group, 58.6% (*n* = 248) made free-text responses. The dominant themes for this group were concerns-about-their-specific-(current)-horse (30.2%); credibility-of-findings (15.7%); and contradictions-and-confusion-with-reported-research (14.9%). Nearly a third of responses from the enthusiast group explicitly mentioned an issue with a specific horse, focusing on challenges to do with: applying theory in practice; navigating conflicting advice and opinions; accessing ‘ethical’ trainers to support them; and combatting ‘old school thinking’ within the wider horse community.

The academic, professional and practitioner groups mostly gave relatively concise responses, such as “The absence of an agreed and standardised nomenclature” (respondent number 54, academic), “Finding reliable and current data on behaviour and treatments for behavioural abnormalities” (respondent number 92, professional) and “Accessibility to peer reviewed information written in easily understood language” (respondent number 81, practitioner). Although there was some variation between them, stakeholders from these three groups emphasised that their main challenges related to the quality of available data and evidence-based training and management programs. 

In contrast, the enthusiast group tended to offer comprehensive details, based on their individual experiences and often related to specific issues with specific horses. Many responses from this group expressed confusion about the breadth of often-conflicting advice available and the difficulties of differentiating between these sources, leading many to feel overwhelmed and to question their own actions. For example “An overwhelming amount of information of low quality (including misinformation), making it hard to find quality information about problems I encounter” (respondent number 151, enthusiast) and “Old acceptedd [sic] wisdom, truth based on personal experience rather than evidence, my own lack of knowledge and understanding” (respondent number 122, enthusiast). Enthusiasts often talked about ‘ethical’ interaction with their horses and expressed doubt about their own abilities to embody the kind of ethical practice to which they aspire. For example, “So the ONE problem is not having a big enough personal experience bank to deal with issues and not being able to access professional opinions I trust to help me deal with any issues” (respondent number 407, enthusiast). 

## 4. Discussion

It is accepted that providing potential survey respondents with incentives to participate is known to boost response rates [17]. Such incentives can be both conditional (supplying contact details, for example) and unconditional (engaging with the survey). The current results support the use of incentives because significantly more respondents indicated that at least one of the motivational items offered was appealing than who nominated none of the items. Furthermore, when offered the choice of three tools, the E-BARQ, *horselogbook*, or the *Share-&-Compare* graph, only 5.9% of practitioners and 9.5% of enthusiasts (see Table 1), selected E-BARQ as the most useful tool. Motivating equine industry stakeholders with individualised feedback, such as a comparison graph specific to their own horses, should also boost respondent completion rates. 

Despite developments in understanding about equine training and welfare that reflect several decades of study, the dissemination of results from empirical studies, and thus their ability to forge change, has been limited. Equitation science [18] and the use of learning theory in training [19] is now well-represented in the literature. However, many coaches and instructors continually fail to grasp the core concepts, thwarting progress at a grassroots level [20,21]. Our findings indicate that this unwelcome state reflects problems with both accessing such information and interpreting and differentiating among different sources of evidence. In fairness, these obstacles to learning contribute to the difficulties that all groups experience. Our results reveal that practitioners and enthusiasts, who do not have institutional access to journals and may lack the experience required to discern the credibility of sources of information, experience particular difficulty applying equitation science and learning theory to their everyday practice. 

Better horse management and training practices increase the prevalence of desirable behaviour and positive welfare outcomes [3,4]. While equitation science has elucidated, incorporated and extended what defines good practice, it will achieve widespread change only when stakeholders understand and assimilate its principles into every-day interactions with their horses. Advances will be accelerated by large-scale data on current behaviour, training and management practices, as they apply to various disciplines, breeds and countries. To define and incorporate good equine training, mental state, housing and behaviour, we need to monitor these parameters over time with a validated tool, such as E-BARQ.

Feedback from items such as the *Share-&-Compare* graph and the *horselogbook* are particularly important for horse owners because horses are large and potentially dangerous animals [22]. Our results demonstrate the desire of horse owners to have a tool to monitor behaviour, as practitioners (49.7%) and enthusiasts (34.5%) selected the *horselogbook* as the most useful item. The *Share-&-Compare* graph enables respondents to benchmark their horse’s behaviour, which was considered as the most useful item for 24.7% of enthusiasts. With an increased ability to monitor and benchmark horse behaviour, we expect to see corresponding increases in rider safety and horse welfare.

Equine and equitation science have been gathering data via online surveys for more than 20 years but never on the scale offered by E-BARQ and never before have researchers attempted to motivate users to engage with such a tool or encouraged multiple longitudinal submissions of data on focal animals to monitor respondents’ own horses’ progress. Our results are encouraging in terms of incentivising respondents to engage with the project. However, the need for improved dissemination of findings also became apparent. The peer-reviewed reference library was selected as the most useful tool by professionals 29.3% of the time while accessibility-of-research-findings was cited by each stakeholder group as a major challenge. Lack of accessibility was referred to by 27.5% of academics and professionals, 33.8% of practitioners and enthusiasts questioned the credibility-of-findings (15.7%) and reported the research-confusing (14.9%).

As seen in our qualitative results, there were also some shared concerns across all stakeholder groups to do with research scale and design (in particular, small sample sizes and difficulties in comparing across studies) and accessing what evidence is available, which tends to be very fragmented and often inaccessible to those without university institutional access to peer-reviewed journals. A central repository to at least reveal where peer-reviewed and evidence-based (and therefore credible) information could be found would be beneficial for all stakeholder groups. However, there were also noticeable differences in emphasis among the groups that reflects their different concerns and interests in research and learning about horse behaviour and management and which will then affect their engagement with E-BARQ. E-BARQ’s capacity to provide a level of standardised terminology and measurements, in addition to a large (and growing) sample size, is expected to be welcomed by all groups.

The qualitative responses from the enthusiast group revealed widespread confusion, doubt and anxiety and point to the need for guidance on evidence-based approaches to horse management and training that can help maximise welfare. E-BARQ has the potential to offer some of this reassurance and guidance to enthusiasts by enabling them to compare their own horse’s behaviour over time and with the wider horse population, helping them understand what can be considered ‘normal’ and what areas may require additional training and support. This level of feedback will be important in encouraging enthusiasts to remain engaged with E-BARQ over the long-term.

These results highlight the extant inadequacies in the dissemination of findings. Prioritising publication in open-access journals will greatly assist with this, building that much-needed bridge between theory and practice. E-BARQ has the potential to further disseminate research results by involving stakeholders as a citizen science project and findings arising from E-BARQ data will preferentially seek publication in open-access journals.

Arguably, the primary appeal of a *Share-&-Compare* feedback graph is to benchmark one’s own horse against others without the costs, effort and risks of competition. The offer of such feedback encourages respondents to return to the questionnaire to update their results at six-monthly intervals. This not only provides researchers with longitudinal data but makes respondents more cognisant of longitudinal trends in their horses’ training, management and resulting behaviour and thus, one might hope, increasingly likely to remediate any deficits in training and management. Respondents’ choice of feedback and tracking tools demonstrates the desire of horse riders and owners to benchmark and monitor their horses over time.

The current results reveal which of the offered motivational items appeal to each stakeholder group. While the *horselogbook* received a higher desirability rating than the *Share-&-Compare* graph, we are currently offering the *Share-&-Compare* graph and are prioritising resources for the *horselogbook* in the future. The two main reasons for this decision are because the *Share-&-Compare* results appear instantly for participants, and, unlike the *horselogbook* option, they negated the need to request for any further data input from participants.

The *Share-&-Compare* graphs give scores in 13 categories [trainability, rideability (for ridden horses only), boldness, handling compliance, working compliance, easy to stop, forward going, human social confidence, non-human social confidence, novel object confidence, touch sensitivity, easy to load and independence]. These categories allow users to identify, at a glance, those areas where their horse might benefit from extra attention or training, thus encouraging the uptake of increased knowledge and improved practice such as the use of combined reinforcement and the elimination of reliance on positive punishment [23] (https://www.e-barq.org/). For example, a horse deemed to be ‘disrespectful’ is likely to be punished [4,24], whereas a horse with a relatively low score on the E-BARQ human-social confidence scale may benefit directly from confidence-building exercises, properly ‘shaped’ training methodologies and social enrichment opportunities with conspecifics. Importantly, covert associations between specific behaviours and deficits or deprivations in the horse’s life may become apparent, not only to practitioners and enthusiasts, but also to researchers.

The current findings show that the most popular incentive for participation was a digital logbook application that would allow users to enter data on their horses’ training, management and behaviour, as frequently as they wish. This result is driving our development of a smartphone-compatible *horselogbook* application that will contain multiple choice, checkbox, frequency and full text question types and encourage riders and coaches track their horses’ management and training progress and could help them understand which training techniques demonstrably work for their horse. The *horselogbook* application will be similar in design and functionality to the University of Sydney’s dog-care and management tracking tool, *doglogbook* (www.doglogbook.com) [25]. It is anticipated that equine data from the *horselogbook*, which are linked to the horses’ unique E-BARQ identification number, will be available to researchers in future. The addition of a user-controlled consent feature will allow users to share their horse’s data with coaches, instructors, veterinarians or any other third party that the users nominate.

This small study had a number of limitations, which we hope will provide guidance to future researchers. Respondents were forced to identify with one of the four stakeholder groups, because the researchers were aiming to collect data from each group. However, this decision may have caused some sampling bias, as many respondents can belong to each of the four groups. In future studies, the authors would advise allowing respondents to select multiple stakeholder groups. With the current study, once respondents had assigned themselves into a stakeholder group, they were offered motivational items based on that choice and described in language appropriate to that group. The difference in language used, the simplified and broad terms with which the items were described, and the single forced choice of those items, made statistical analysis challenging. Future researchers should consider providing respondents with a full description of each item, assessing their understanding of the terms used and allowing stakeholder groups to rank their perceived usefulness.

Distribution of the link to the survey instruments discussed here was limited to social media, including Twitter, LinkedIn, FB and Instagram, in addition to two electronic newsletter lists. While potential respondents not on these lists or non-social media users were unlikely to access the link, this type of distribution is most commonly used when exploring industry practice [26]. This online inquiry may have suffered from sampling bias as it is possible that our respondents were likely more engaged with equitation science than the general horse community.

## 5. Conclusions

This study revealed that equine stakeholder groups greatly value data that benchmark their horses’ behaviour against that of the general equine population. They also see merit in being able to log their training and management via an app and monitor their horses’ behaviour over time. The study further revealed the particular challenges horse enthusiasts and practitioners face when attempting to gather information on training, management and behaviour. We conclude that offering the motivational items in addition to the full E-BARQ questionnaire and choosing open-access publications where possible for the dissemination of results, should bridge the knowledge gap, while encouraging potential users to engage with the tool.

## Figures and Tables

**Table 1:** The distribution of respondents from four stakeholder groups that were offered tools based on their anticipated appeal (and percentage of that stakeholder group) that indicated which incentive item, or ‘none’, they would find most useful, in a forced single-choice answer. N/A indicates that tool was not offered to that stakeholder group.

Incentive Item	Academics *n* = 83 (11%)	Professionals *n* = 78 (10.5%)	Practitioners *n* = 137 (18.3%)	Enthusiasts *n* = 449 (60.2%)
E-BARQ database	*n* = 36(43.9%)	*n* = 37 (45.1%)	N/A	N/A
E-BARQ questionnaire alone	*n* = 35(42.7%)	N/A	*n* = 9 (5.9%)	*n* = 54 (9.5%)
*Horselogbook*	N/A	N/A	*n* = 76 (49.7%)	*n* = 195 (34.5%)
Peer-reviewed reference library	N/A	*n* = 24 (29.3%)	N/A	N/A
*Share-&-Compare* graph	N/A	*n* = 17 (20.7%)	*n* = 36 (23.5%)	*n* = 140 (24.7%)
None of the above	*n* = 11 (13.4%)	*n* = 5 (6.1%)	*n* = 32 (20.9%)	*n* = 179 (31.6%)

A χ^2^ test showed that the perception of at least one tool as useful varied among respondent groups (χ^2^ = 35.105, df = 3, *p* < 0.001). Enthusiasts were least likely to perceive an offered tool as useful, significantly less so than practitioners (adjusted *p* = 0.023), Academics (*p* = 0.006) and professionals (*p* < 0.001). Practitioners were also significantly less likely to perceive an offered tool as useful, than were professionals (*p* = 0.042).

## References

[1] Hausberger M., Gautier E., Biquand V., Lunel C., Jego P. (2009). Could Work Be a Source of Behavioural Disorders? A Study in Horses. PLoS ONE.

[2] Odberg F., Bouissou M.-F. (1999). The development of equestrianism from the baroque period to the present day and its consequences for the welfare of horses. Equine Vet. J..

[3] McGreevy P.D., Berger J., De Brauwere N., Doherty O., Harrison A., Fiedler J., Jones C., McDonnell S.M., McLean A., Nakonechny L. (2018). Using the Five Domains Model to Assess the Adverse Impacts of Husbandry, Veterinary, and Equitation Interventions on Horse Welfare. Animals.

[4] International Society for Equitation Science (ISES) (2017). Position Statement on the Use/Misuse of Leadership and Dominance Concepts in Horse Training. https://equitationscience.com/equitation/position-statement-on-the-use-misuse-of-leadership-and-dominance-concepts-in-horse-training.

[5] Loftus L., Marks K., Jones-McVey R., Gonzales J.L., Fowler V.L. (2016). Monty Roberts’ Public Demonstrations: Preliminary Report on the Heart Rate and Heart Rate Variability of Horses Undergoing Training during Live Audience Events. Animals.

[6] Veen I., Killian D., Vlaminck L., Vernooij J., Back W. (2018). The use of a rein tension device to compare different training methods for neck flexion in base-level trained Warmblood horses at the walk. Equine Vet. J..

[7] Dashper K. (2014). Tools of the Trade or Part of the Family? Horses in Competitive Equestrian Sport. Soc. Anim..

[8] Hemsworth L.M., Jongman E., Coleman G., Ellen J. (2015). Recreational horse welfare: The relationships between recreational horse owner attributes and recreational horse welfare. Appl. Anim. Behav. Sci..

[9] Hecht J., Rice E.S. (2015). Citizen science: A new direction in canine behavior research. Behav. Process..

[10] Townsend L., Dixon L., Chase-Topping M., Buckley L. Who’s walking who? The relationship between pulling on lead and pet dog welfare in the UK and Ireland. Proceedings of the Advances in Animal Welfare Science VII: UFAW Animal Welfare Conference, University of Birmingham.

[11] Todd Z. (2018). Barriers to the adoption of humane dog training methods. J. Vet. Behav..

[12] Birke L. (2008). Talking about Horses: Control and Freedom in the World of “Natural Horsemanship”. Soc. Anim..

[13] Buhrmester M., Talaifar S., Gosling S.D. (2018). An Evaluation of Amazon’s Mechanical Turk, Its Rapid Rise, and Its Effective Use. Perspect. Psychol. Sci..

[14] Duda M.D., Nobile J.L. (2010). The Fallacy of Online Surveys: No Data Are Better Than Bad Data. Hum. Dimens. Wildl..

[15] O’Reilly-Shah V. (2017). Factors influencing healthcare provider respondent fatigue answering a globally administered in-app survey. PeerJ.

[16] Singer E., Vannette D., Krosnick J. (2018). Survey Incentives. The Palgrave Handbook of Survey Research.

[17] Young J., O’Halloran A., McAulay C., Pirotta M., Forsdike K., Stacey I., Currow D.C., Forsdike-Young K. (2015). Unconditional and conditional incentives differentially improved general practitioners’ participation in an online survey: Randomized controlled trial. J. Clin. Epidemiol..

[18] McGreevy P.D. (2007). The advent of equitation science. Vet. J..

[19] McGreevy P.D., McLean A.N. (2007). Roles of learning theory and ethology in equitation. J. Veter Behav..

[20] Warren-Smith A.K., McGreevy P.D. (2008). Equestrian Coaches’ Understanding and Application of Learning Theory in Horse Training. Anthrozoös.

[21] Brown S.M., Connor M. (2017). Understanding and Application of Learning Theory in UK-based Equestrians. Anthrozoös.

[22] Hawson L.A., McLean A.N., McGreevy P.D. (2010). The roles of equine ethology and applied learning theory in horse-related human injuries. J. Vet. Behav..

[23] McLean A., McGreevy P., Christensen J.W., ISES (2018). Principles of Learning Theory in Equitation.

[24] McGreevy P.D., McLean A.N. (2009). Punishment in horse-training and the concept of ethical equitation. J. Veter Behav..

[25] McGreevy P., Starling M., Payne E., Bennett P. (2017). Defining and measuring dogmanship: A new multidisciplinary science to improve understanding of human-dog interactions. Vet. J..

[26] Jaqueth A.L., Hathaway M., Catalano D.N., Linders N.C., Mottet R., Martinson K.L. (2019). Using Web-Based Surveys to Explore Equine Industry Practices and Future Research Needs. J. Equine Veter Sci..

